# Opiate Use Patterns Among Collegiate Athletes

**DOI:** 10.7759/cureus.31152

**Published:** 2022-11-06

**Authors:** Caitlin M Rugg, Jonathan W Cheah, Rock P Vomer, Brian Lau

**Affiliations:** 1 Department of Orthopedic Surgery, Kaiser Permanente San Jose Medical Center, San Jose, USA; 2 Department of Orthopedic Surgery, Santa Clara Valley Medical Center, San Jose, USA; 3 Family and Community Health and Orthopedics, Division of Sports Medicine, Duke University, Durham, USA; 4 Orthopedic Surgery, Duke University, Durham, USA

**Keywords:** orthopedic sports medicine, sports medicine, gender, pain management, collegiate athlete, opiate misuse

## Abstract

Objective/Aim: The purpose of this study is to determine the rates of prescribed opiate use and misuse among current collegiate athletes.

Materials and methods: This was an *o*bservational survey study conducted at a single institution; Division I Collegiate Athletics Department. The participants in the study were current Division I Collegiate Student-Athletes. The survey queried athletes’ age, gender, and history of injury or orthopedic surgery before and during college. Athletes were asked about prior opiate prescriptions, length of medication use, and reasons for opiate use.

Results:Of196 student-athlete respondents, the average age was 20.1 years and 62.8% were female. Pre-collegiate orthopedic injuries/surgeries were reported by 45.4% of athletes, of which 40.4% received an opiate prescription. Collegiate orthopedic injuries/surgeries were reported by 28.6% of athletes; 46.4% received an opiate prescription. Fifty-two student-athletes (26.5%) had received an opiate prescription after an orthopedic injury or surgery. The length of opiate use was most commonly 2 weeks or less. Female athletes had a higher rate of collegiate injuries (*P*<0.05) and a nonsignificant trend towards more opiate prescriptions. Among the 26 student-athletes who received collegiate opiate prescriptions, the reasons for taking opiates were most commonly pain (84.6%) and sleep (46.2%). Opiate use outside of prescribed indication was present in 14 athletes (7.1% of the total); 12 were female.

Conclusion:A quarter of collegiate student-athletes had received an opiate prescription due to orthopedic injury or surgery, with a small subset using opiates for non-analgesic functions. Future research should examine risk factors for opiate misuse among collegiate athletes.

## Introduction

Opiate use is on the rise in many populations across the United States over the past two decades [1,2]. Furthermore, the unintended use of opiates is common and may consist of using opiates for recreation, stress relief, sleep, and mood enhancement [3]. Opiate misuse is a rising issue among adolescents, college students, and young adults [4-6]. Additionally, there is a concern for potential misuse among adolescents, high school and colligate.

Orthopedic surgery is a common source of initial opiate prescription for chronic opiate users, and it is important to recognize due to the outsized role that opiate prescriptions have in becoming a gateway to opiate misuse [7-9]. College athletes are at an increased risk for orthopedic surgery relative to their peers [9]. Additionally, athletes may experience pain due to their sports and injuries. In prior literature from the National Institute on Drug Abuse, middle and high school athletes’ use of opioids has been characterized by an incidence of up to 35% [10]. Of retired professional NFL athletes, 26% reported recent use of opioids [11]. Currently, the literature surrounding opiate use in contemporary collegiate athletes recovering from orthopedic injury or surgery is sparse.

The use of opioids among athletes at the high school and college level has not been well-characterized and could be an important period for intervention prior to entering adulthood and professional sports. The purpose of this study was to determine the rates of opiate use and misuse related to a prescription for an orthopedic diagnosis among current Division I NCAA college athletes.

## Materials and methods

This study was a single-institution study and Duke University Health System Institutional Review Board approval was obtained prior to the study. The institution of the investigation participates in NCAA Division I athletics. The school enrolls approximately 6,000 students per year and 650 student-athletes participate annually. The study data were collected and managed by using REDCap (Research Electronic Data Capture) electronic data capture tools and were hosted at the Institution [12,13]. REDCap is a secure, web-based software platform designed to support data capture for research studies, which provides an intuitive interface for validated data capture; audit trails for tracking data manipulation and export procedures; automated export procedures for seamless data downloads to common statistical packages, and procedures for data integration and interoperability with external sources. Student-athletes were eligible to participate if they were active participants in athletics and included both genders and 16 sports. The sports included are listed in Table 1.

**Table 1:** Sports included by gender

Men’s Sports	Women’s Sports	Mixed Gender Sports
Baseball	Softball	Cross Country
Basketball	Basketball	Fencing
Football	Field Hockey	Rowing
Golf	Golf	Swimming & Diving
Lacrosse	Lacrosse	Track & Field
Soccer	Soccer	
Tennis	Tennis	
Wrestling	Volleyball	

A REDCap-based survey was anonymously and electronically distributed to the student-athletes by the certified athletic trainers associated with each sports team. The survey is outlined in Appendix 1. The survey queried the athletes’ age and gender, history of injury, and orthopedic surgery. To protect anonymity and encourage voluntary participation in the survey, student identification was minimized by omitting the type of sport played, year in school, and other demographic identifiers. Athletes were asked about their history of orthopedic injuries/surgeries and opioid use at two-time points, pre-college and during college. These questions asked if they had received a prior opiate prescription, and how long they used opiate medications following injury or surgery. Student-athletes who had received an opiate prescription in and prior to college were asked their reasons for opiate use. The reasons for opiate use included: pain; sleeping; changing the mood or being happy; relaxing, calming down or relieving stress, and to function. Appropriate use was deemed using opiate medication for pain; opiate misuse was deemed using opiate medication for any indication other than pain.

The percentages of athletes who received opiate prescriptions were calculated. Chi-Squared tests were performed to identify statistically significant differences between male and female athletes regarding injury occurrence, opiate prescriptions, and opiate misuse. Comparisons were deemed to be statistically significant using a threshold of P < 0.05. Statistical analyses were performed using SPSS 24 software (IBM Corp., Armonk, NY, USA).

## Results

There was a total of 281 female and 378 male collegiate athletes who received an invitation for the survey. Of the 659 surveys distributed, 196 student-athletes completed the survey for a response rate of 29.7%. The average student-athlete age was 20.1 years. Female athletes made up 62.8% (123/196) of respondents and male athletes made up 37.2% (73/196); this correlated to a 43.8% response rate (123/281) among female athletes and 19.3% (73/378) response rate among male athletes. Demographic, injury, and opiate prescription data are shown in Table 2.

**Table 2:** Student-Athlete Demographics Percentage of athletes using prescribed opiates by length of use and precollegiate/collegiate injury. Rx = prescription.

Variable		n	Percentage	
Total		196	100	
Gender				
	Female	123	62.8	
	Male	73	37.2	
Precollegiate Injury				
	Yes	89	45.4	
	No	107	54.6	
Precollegiate Opiate Prescription			Of injured athletes (n=89)	Of all athletes (n=196)
	Yes	36	40.4	18.4
Collegiate Injury				
	Yes	56	28.6	
	No	130	66.3	
Collegiate Opiate Prescription			Of injured athletes (n=56)	Of all athletes (n=196)
	Yes	26	46.4	13.3
Any Prior Opiate Prescription				
	Yes	52	26.5	
	No	144	73.5	

Pre-collegiate orthopedic injuries/surgeries were reported by 45.4% of athletes, and 40.4% of these athletes received an opiate prescription, which represented 18.4% of all athletes. College orthopedic injuries/surgeries were reported by 28.6% of athletes, and of these athletes, 46.4% reported a related opiate prescription, which represented 13.3% of all athletes. In total, 52 student-athletes (26.5%) reported receiving an opiate prescription after an orthopedic injury or surgery at either time point. Length of opiate use was most commonly less than one week (77.8% pre-collegiate, 69.2% collegiate) or approximately two weeks (19.4% pre-collegiate, 19.2% collegiate) (Table 3). Two collegiate and one pre-collegiate athletes used opiates for around four weeks, and one collegiate athlete used opiates for three months, while none used them for six months. 

**Table 3:** Precollegiate and collegiate injuries/surgeries and opiate prescriptions by gender SA = Student-Athlete. Rx = Prescription. * = P<0.05.

	Female	Female %	Male	Male %	Pearson Chi Square
Precollegiate injury	57	46.3	32	43.8	0.733
Precollegiate opiate prescription	24	19.5	12	16.4	0.863
Precollegiate Opiate prescription length of use					
<1 week	18	14.6	10	13.7	0.825
2 weeks	5	4.1	2	2.7	
4 weeks	1	0.8	0	0	
3 months	0	0	0	0	
6 months	0	0	0	0	
Collegiate injury	43	35.0	13	17.8	0.033*
Collegiate Opiate rx	21	17.1	5	6.8	0.031*
Collegiate Opiate prescription length of use					
<1 week	15	12.2	3	4.1	0.12
2 weeks	4	3.3	1	1.4	
4 weeks	2	1.6	0	0	
3 months	0	0	1	1.4	
6 months	0	0	0	0	
Collegiate misuse**	12	9.8	2	2.7	0.065
* denotes p<0.05 ** defined as us of opiate medication for an indication other than pain					

When examining injury and opiate prescription by gender, there were near equivalent rates of pre-collegiate orthopedic injuries/surgeries and opiate prescriptions for male and female student-athletes (Table 3). However, at the college level, twice as many female respondents experienced orthopedic injury or surgery (Female: 35%; Male: 17.8%, p=0.03), and three times as many female respondents reported receiving an opiate prescription (Female: 17.1%; Male: 6.8%, p=0.03).

Of the 26 student-athletes who received opiate prescriptions during college, the athlete reported reasons given for taking opiate medication were most commonly pain relief (n=22, 84.6%) and sleep (n=12; 46.2%). All athletes were initially prescribed opiates for pain control. Athletes who reported opiate misuse taking opiates were present in 14 athletes (7.1% of all athletes) and are detailed in Table 4. Of these 14 athletes, 12 were female (representing 9.8% of all female athletes) and two were trended towards being more likely to have received an opiate prescription in high school (5/14, 35.7%); the rate of misuse for athletes who did not have a high school prescription was much lower (9/160, 5.6%) (p=0.082).

**Table 4:** Reasons for opiate use following collegiate prescription Percentage of student athletes who received a collegiate opiate prescription that indicated each reason for opiate use.

	n	Percentage of Athletes (n=26)
For pain	22	84.6
To sleep	12	46.2
To function	3	11.5
To relax, calm down, or relieve stress	3	11.5
To change mood or be happy	3	11.5
Any non-pain use*	14	53.8
Total	26	100
*Non-pain use defined as any of the following: to sleep; to function; to relax calm down, or relieve stress; to change mood or be happy.		

## Discussion

In a survey of current NCAA student-athletes, we found that a quarter has received an opiate prescription before or during college for an orthopedic injury or surgery. The vast majority of athletes used opiates for two weeks or less. This study found female athletes in this study were over twice as likely to have had an orthopedic injury/surgery and receive an opioid prescription during college. While the pain was the most common reason for opiate use among college athletes with an opioid prescription, over half of the college athletes with an opioid prescription during college reported a non-indicated use of opioids. This behavior was more common in women, present in approximately 10% of female respondents.

This is one of the first studies to examine opiate use among collegiate athletes. Prior studies have focused on the college student population at large or the general population. McCabe et al. surveyed collegiate students from 2003 to 2013 with regard to medical and non-medical use of prescription drugs, including opioids, and found that approximately one-fifth of students had used a prescription drug at any point [14]. The rate of lifetime opioid use dropped over the time period and ranged from 8.8% to 16%. This rate of use is lower than the rate of opioid use seen in the present study. One plausible explanation is that collegiate athletes as a group have experienced more injuries and resulting pain compared to non-athletes. While historically sports participation among youth was thought to be protective against risky behavior such as drug and alcohol use, in actuality the literature is mixed [15-17]. A 2010 review of 34 articles on alcohol and drug use among collegiate athletes demonstrated reduced cigarette use and illicit drug use (including opioids) but increased use of alcohol [18]. However, more recent studies with a focus on opioids raise a concern about opiate misuse in young athletes. A 2013 survey study, examined the rates of opiate use and disuse among college students who had a history of interscholastic sports participation versus those who did not [19]. The authors found that college students who had participated in interscholastic sports in high school were more likely to have repeated lifetime medical use of opioids (three or more episodes) and were more likely to have been approached about diverting opioids than non-athletes. Furthermore, survey studies of adolescent athletes have identified participation in high-injury sports and male gender as risk factors for pain medication misuse [19,20]. In a systematic review of opiate use in athletes, Ekhtiari et al. described rates of opiate use in high school athletes as 28%-46%, which is higher than the rate of 18% described in this study [21]. This may be due to the method of the survey. In the present study, the goal was to determine opiate use patterns in athletes who had a history of orthopedic injury or surgery and to determine the impact of those events on opiate use patterns in athletes. However, in other studies, all high school athletes were surveyed about opiate use. The higher rates reported in the Ekhtiari study may also be attributed to recall bias; athletes in the present study were surveyed retrospectively and may not have had an accurate recollection of their post-injury or post-surgery pain medication regimen. As the landscape and epidemiology around opiate use and misuse change, contemporary studies must include this vulnerable population.

The role of gender in opiate misuse has been studied on a population level. Acute and chronic pain are more common among women, and women are more likely to receive an opiate prescription than men [22]. As opiate prescriptions are a common entrance to opiate misuse, one would expect an increased prevalence of opiate misuse among women. In fact, a national survey from 2002 to 2014 found that new opiate dependence was more common among adolescent and young adult females than males [23]. In the military population, Anderson et al. demonstrated a greater chance of prolonged opiate use after anterior cruciate ligament reconstruction for female patients [24]. Furthermore, risk factors for prolonged opiate use after shoulder arthroscopy include female gender, prior substance abuse, mental health or mood disorder, and chronic pain diagnosis [25].

In addition to being more likely to sustain injury and receive an opiate prescription in college, there was a non-significant trend towards women being more likely to misuse opiates for a non-pain indication than male athletes. These indications included “to sleep,” “to function,” “to relax, calm down, or relieve stress” and “to change the mood or be happy.” It is common for collegiate athletes to experience difficulty coping after an orthopedic injury or surgery, and athletes with a prior sports injury are more likely to have a diagnosis of anxiety [26,27]. Furthermore, stress is a well-known risk factor for addictive behavior and substance abuse [28,29]. Gender may also impact how an athlete copes with injury, their post-injury psychological well-being, and their return to play readiness [30]. While only a small percentage of athletes indicated this type of misuse, it is a relatively similar rate to the published rates in the general public. At the population level, a study of the 2016-2017 National Surveys on Drug Use and Health demonstrated that about 12% of respondents reported opiate misuse, the majority by using their own prescriptions. When stratified by gender, men were more likely to misuse prescription opiates, but women who misused opiates were more likely to have a mental health disorder [31].

The authors recommend that for all collegiate athletes, maximizing non-opiate modalities for pain should be attempted, as well as addressing comorbid issues such as sleep, coping, and underlying mental health disorders. A focus on multimodal pain management has become increasingly recognized to help reduce opiate use, and these techniques can be applied to collegiate athletes as well [32]. Non-opiate medications such as acetaminophen and non-steroidal anti-inflammatories (when appropriate) are used regularly and are an excellent option for transition after opiates have been discontinued. In the training room, ice, compression, elevation, and transcutaneous electrical nerve stimulation were used to reduce swelling and control pain as well. Education of student-athletes about prompt discontinuation of opioid pain medication and home application of the above techniques is emphasized. Finally, enhancing social support around injured athletes can help diminish perceived stress after an injury [33]. At our institution, team athletic trainers attend medical appointments with the athletes. Sports psychology referral is available to all athletes but is emphasized for athletes with a season-ending injury or surgery. Further research is needed to determine if these types of interventions truly reduce the length of time of opiate use and the incidence of misuse in the collegiate athlete population.

Unique to this study was determining the reasons for opiate misuse among collegiate athletes. While pain was the most common indication for use among athletes, nearly half also indicated using opiates for sleep. Disrupted sleep has been characterized by a number of orthopedic conditions [34-37]. Animal and human studies have shown that sleep deprivation can have negative effects on stress corticosterone levels and pain perception [38,39]. This is of great concern in our athletic population as it has been shown that college athletes who experience sleep deprivation have reduced physical performance, coordination, and response times [40].

While orthopedic surgery can generally improve disrupted sleep, its improvements occur in a delayed fashion that ranges from six weeks to six or more months after surgery [34-37] In acute orthopedic injuries that have significant pain that leads to disrupted sleep, there may be an opportunity to intervene on this with directed sleep therapy treatments. Cognitive and behavioral interventions that address stress management, relaxation practices, stimulus control, sleep hygiene, and exercise can improve sleep quality and duration in adults [37,41]. Non-pharmacological sleep interventions, such as extending sleep duration and improving sleep hygiene in athletes, have beneficial effects of reduced fatigue, enhanced cognitive reaction time, improved mood, and optimized athletic peak performance [42]. The use of non-pharmacological interventions has been shown to be more efficacious for promoting long-term sleep health when compared to pharmacological treatments [43]. Melatonin remains an attractive pharmacologic option due to its favorable side-effect profile and minimal drug-drug interactions. While studies examining melatonin use after orthopedic surgery are sparse, melatonin has been shown to improve psychomotor performance in young athletes in the setting of sleep disruption [44,45].

Limitations

This was a single-institution study in which all athletes participating in a single year were surveyed; as such, no a priori power analysis was performed due to the design. Furthermore, the small sample size only allowed for limited statistical analysis. Athlete demographic information such as sport played, amount of total opiate consumed, and year in school was not collected to protect the anonymity of the athletes, but this limited the ability to examine further risk factors for opiate use. Athlete information was collected using an unvalidated questionnaire with closed-ended questions. This process was selected because the goal of the study was to identify what if any patterns of opiate misuse existed in the colligate athlete population. In follow-up studies that include multiple centers, a validated questionnaire could be used to improve the study. Only athletes with a history of orthopedic injury or surgery were asked about opiate use, with the goal of identifying athletes who received opiates directly as a result of an orthopedic diagnosis. However, athletes who used opiates outside of those indications were not identified. In this study female athletes had a much higher response than male athletes. This difference in response rate could identify higher use of opiates or demonstrate a better survey response rate in female athletes. Both of these points should be further investigated in follow-up studies. Despite these limitations, this study adds to the small body of literature examining the important topic of opiate use and misuse in a population that is exposed to opiates at a young age.

## Conclusions

Based on the findings from this study, the authors recommend a continued focus on a multidisciplinary approach for post-injury and post-operative pain management in collegiate and high school athletes. Non-opiate pharmacologic and non-pharmacologic modalities and educational interventions should be maximized to reduce need for opiate prescriptions and use. Recognizing and addressing concurrent sleep disruption, stress, and mood disorders are important to minimize opiate misuse in the collegiate athletic population. Opiate prescriptions and misuse were more common among female athletes, and the role of gender is an area for further investigation.

## Appendices

Pain Medication Use Survey

Please complete the survey below.

Thank you!

This short survey will help provide us with information regarding opioid pain medication use among high-level college athletes after orthopaedic surgery.

[Page1]

The information collected will be completely anonymous and used for research purposes only. The information collected will help guide your medical team when prescribing these medications.

This survey will NOT affect your eligibility to participate in NCAA sports.

By clicking below, I agree to participate in the following anonymous survey. 

I agree Disagree

What is your age in years?

__________________________________

What is your gender?

Male Female

[Page 2]

We will now be talking about opioid pain medication use BEFORE COLLEGE.

Opioid pain medications include medications known as: morphine, oxycodone, hydrocodone, norco, Percocet, dilaudid, or hydromorphone.

Prior to college, have you injured a bone, joint or ligament that required an orthopedic evaluation and/or surgery?

Yes No

After you had orthopedic injury and/or surgery were you prescribed opioid pain medications such as: morphine, oxycodone, hydrocodone, norco, percocet, dilaudid, or hydromorphone?

Yes No

During this time, do you remember for how long you used opioid pain medications? (choose one)

Less than 1, week Approximately 2 weeks, Approximately 4 weeks, Approximately 3 months, More than 6 months

[Page 3]

We will now be talking about opioid pain medication use DURING COLLEGE.

Opioid pain medications include medications known as: morphine, oxycodone, hydrocodone, norco, Percocet, dilaudid, or hydromorphone.

During college, have you injured a bone, joint or ligament that required an orthopedic evaluation and/or surgery?

Yes No

After you had orthopedic injury and/or surgery, were you prescribed opioid pain medications such as: morphine, oxycodone, hydrocodone, norco, percocet, dilaudid, or hydromorphone?

Yes No

During this time, do you remember approximately for how long you used opioid pain medications?

Less than 1 week, Approximately 2 weeks, Approximately 4 weeks, Approximately 3 months, More than 6 months

After your orthopaedic surgery, did you use opioid pain medications for the following reasons: (Choose all that apply)

To function To change mood or be happy To relax, calm down, or relieve stress To sleep For pain
